# Case of Suspended Animation by Hanging

**Published:** 1806-12

**Authors:** Robert John Thornton

**Affiliations:** Lecturer on Medical Botany at Guy's Hospital, with Observations on the Modes of Treatment, and recommending a new and easy Process for Recovery


### CASE *of* SUSPENDED ANIMATION *by* HANGING, *by* ROBERT JOHN THORNTON, M. D. *Lecturer on Medical Botany at Guy's Hospital, with Observations on the Modes of Treatment, and recommending a new and easy Process for Recovery.*

MR. B———, a tradesman in Duke-street, Manchester-square, from causes which I need not enter into, resolved upon self-destruction. He deliberately retired to a garret in his house, and wrote down his causes of distraction, which he left upon the table. He sent the maid-servant down stairs, and leaped, as he thought, into eternity.

It being the top of the house, and Sunday, the rest of the family at church, his wife, child, and servant below stairs, no one heard his struggles, and he was full twenty minutes before the wife entered the apartment, saw her husband suspended, and to all appearance dead. He was cut down, and every assistance sent for in the neighbourhood. When I arrived, all pulsation had ceased, and animation appeared fled. In all cases of apparent death, time presses, and the urgency of the case demands all possible expedition. The grand question is, what is *first* to be done, and by what means? Then it may be right to premise, that I consider hanging and drowning as the same effect, but only produced by a different cause. Both may be thus defined, "a stop put to the actions of life in the body, without any irreparable injury to any vital organ; but it is requisite to put the animated machinery into action in a given time, or the power of action will be irrecoverably lost." Cullen, Boerhaave, Mr. White, &c. &c. have, on the contrary, considered suspension and drowning as extremely different; for hanging, they represent as destroying by inducing *apoplexy*. This also is the common, and a very natural opinion; but I shall here prove it to be erroneous. The following experiment was long since made by Professor Monro, at Edinburgh. A dog was suspended by the neck with a cord; in a few minutes he ceased to struggle. The same experiment, exactly, was tried upon another dog, but an opening was previously made in the wind-pipe below the cord, so as to admit of air being forced into his lungs. In this state he was kept alive three quarters of an hour, when the cord was shifted below the opening into the wind-pipe, so as to intercept the ingress of air into the lungs, and the animal died in a few minutes. Upon examining the head, there was found *no rupture of vessels* in the brain. To prove this point more clearly, I shall relate a still more decided experiment by the veterinary Professor, Mr. Coleman. The carotids, or arteries, which carry blood to the head, may be secured without soon materially destroying the animal functions. This operation was first done, and then the animal was hanged, and he died in a few minutes. Upon the brain being examined, there was found a *less congestion of blood* than usual, and therefore *no apoplexy*. In *apoplexy* the irritability continues several hours, whilst in drowning or hanging, the animal functions are abolished in a few minutes. In *apoplexy*, respiration, together with the action of the heart and arteries go on, and the pulse often vibrates more forcibly than in health. In hanging or drowning respiration is suppressed, and the pulse obliterated. In *apoplexy* there is the stertor *apoplecticus* very distinct; this is not to be discovered in hanging: and, lastly, after recovery from *apoplexy*, the subject is generally paralytic; whereas no such event follows recovery from suspension; and where death ensues, the appearances in the brain, in the two instances, are entirely *toto cœlo* different. I press this distinction forward, as the proximate cause being ascertained leads to the right mode of treatment. This *proximate cause* I have sufficiently proved to be, in both hanging and drowning, a stoppage of air to the lungs, by which the venous blood ceases to imbibe

*oxygen*,

*oxygen,* and hence the heart, unstimulated by *oxygen,* ceases to contract, the arteries to vibrate, and hence the whole machine, although sound and intire in every part, yet on a sudden, like a clock, whose pendulum is stopped, remains entirely at rest.

In the watch, if we move but the pendulum, the wheels are immediately put into motion, the clock again correctly marks its hours and minutes as before: so likewise in the animal machine, if the blood can but be influened by *oxygen,* the heart recovers its action, the brain its energy, and the nerves their sensibility; such is the wonderful harmonious consent of parts!

From this privation of *oxygen* in drowning and suspension, we can now explain why the blood grows dark, the lips and countenance livid, and why the body quickly loses its animal heat. That the *motion* of the *heart* depends upon the *air* thrown *into the lungs,* was established by the illustrious Hook, a century ago, before the Royal Society. He laid open the thorax of a dog, cut away the ribs and diaphragm, and removing the pericardium, he kept the animal alive above an hour by means of a pair of bellows. It was observed, that as often as he left off blowing, and the lungs were collapsed, the dog fell into convulsive motions, and revived again upon renewing the blast, and the heart began afresh to beat. John Hunter repeated the same experiment, and hence concludes, as with still-born children, in suspended animation, the *first* thing is *to force air into the lungs to restore the heart's action.* For this purpose, John Hunter invented a pair of bellows of such a construction, that by one action fresh air is thrown into the lungs, and by another it is pumped out again, to imitate artificial breathing. This invention may be seen at Savigny's. But in this case no such instrument could be procured, I therefore requested a common pair of bellows, and by inserting the nozzle up one nostril, while the mouth and opposite nostril were closed, by a forcible pressure, the lungs were expanded with commou air.

We are now come to the next process. The heart or nerves are to be roused into action. Volatiles, especially aromatic vinegar, &c. are recommended by John Hunter; but these are not always at hand. I requested the servant, at the time she brought the bellows, to bring some *mustard* and *boiling water.* As soon as the lungs were dilated, some mustard was put up the nostril and into the mouth, and a cloth dipped in boiling water, that is water scalding hot, was put over the region of the heart and thorax.

 This

This had a double effect; it stimulated the heart by exciting the nerves, and by imparting heat, gave it more susceptibility to be acted upon. Some years ago, says Dr. Gardiner, the heart and part of the large vessels of a turtle were removed, with a view to examine the structure of these parts, and the circulation of the blood of that animal. Having wiped off the blood and other moisture, the heart was wrapped up in a handkerchief; but engagements in the way of my profession obliged me to postpone my curiosity till about six or seven hours after it was cut out. When I examined it, there appeared not the least signs of life; but by putting it into water, nearly *milk warm,* it acquired a tremulous motion; laying it on the table, and pricking it with a large needle, it palpitated several times. The palpitations were renewed, as often as the needle was pushed into its substance, until it become *cold,* when it seemed to be insensible to every stimulus. But after *warming* it again in the water, it recovered its irritability, and repeated its palpitations on the application of the needle. Though no movement could be excited in it by any stimulus when cold, yet it moved several times after being placed in the *warm* water. This proves the necessity of *heat* for maintaining the full powers of the contractile living fibre.

For the purpose of stimulating the heart, *electricity* is recommended by John Hunter. But this is often as unattainable as his bellows.

Immediately upon the inflation of the lungs, and the application of the stimulus of boiling water to the chest, our patient gave signs of returning life, by an apparently painful struggle; his eyes dreadfully rolled, his countenance became distorted, and the struggles of convulsive nature ensued.

The legs were next rubbed with mustard, as also the thighs and arms, he was also continually blown upon by the bellows, raised upon the bed. In order to oxygenate the air the more, and stimulate the olfactory nerves, six quarts of *vinegar,* in fine sprays from an hearth broom, were dispersed over the apartment. The acid principle of the vinegar is the product of *oxygen,* the reader already knows. In order to keep up the nice sympathy which exists betwixt the *lungs, heart,* and *stomach,* a cordial of very weak *brandy and water* was forced into the stomach, sweetened with sugar.

In time of health, *cordials,* as they are called, on being received into the stomach, presently manifest their enlivening livening effects; even long before they can be supposed to enter the lacteals and pass to the heart, their stimulus is difused to the most remote part of the system. The rationale of this appears to be as follows. *Alcohol* has an high affinity for *oxygen,* and by combustion it has been discovered, that sixteen ounces of the former being burnt, produces eighteen ounces of water, which we know to be composed of *hydrogen* and *oxygen.* From this rapid combustion much *caloric* or *heat* is extracted. In order to give a distinct idea of the exact quantity, one pound weight of *hydrogen* melted 295 pounds of ice, whereas in similar circumstances, a pound of wax candles only melted 133 pounds. Not only a great degree of animal heat therefore arises from the union of *hydrogen* and *oxygen,* but the nerves which supply the heart and stomach, the par vagum, are therefore stimulated, and act by sympathy, as snuff stimulating the olfactory nerves produces sneezing, or spasm of the abdominal muscles; and it is also found, that the blood by possessing *hydrogen* becomes more attractive of the *oxygen.* Dr. Withering, writing to Dr. Beddoes, says, " The experiments you wished for have been in part made. The late ingenious Mr. Spalding, who did so much in improving and using the diving-bell, and had practised with the greatest success for many years, was a man of nice observation, and had he not fallen a sacrifice to the negligence of drunken attendants, would have himself informed you of the circumstance. He particularly informed me, that when he had eaten animal food, or drank fermented liquors, he *consumed the air in the bell faster* than when he lived upon vegetable food, and drank only water. Many repeated trials had so convinced him of this, that he constantly abstained from the former diet, whilst engaged in diving."

John Hunter recommends " the conveyance of *some stimulating substance* into the stomach, to rouse this seat of universal sympathy. This operation should be performed with all possible expedition for fear of inducing sickness."

What this *stimulating substance* should be, he has not informed his readers. The mode of conveyance, is by means of a spoon pressing down the tongue, the patient being partly elevated. Among the class of internal stimulants are hartshorn, rum, brandy, and usquebaugh, which are powerful stimulants. But here it should be remembered, that the cessation of the actions of parts, predispose them to be affected by lesser stimuli, and thrown into inordinate

 action action by any strong stimulus. Thus, in a frost-bitten limb, the actions have ceased but the power remains. Heat only is wanting to call this power into action. But this must be gradually applied, or the highest inflammation will ensue, ending in mortification. So with those starved nearly to death by hunger. Thus may the salutary efforts of nature be overpowered by the officiousness of art, a circumstance we may have frequent occasion to observe with regret. Thus, if our stimulants are too potent, they may prove destructive, by soon exhausting the living fibre. We must recollect here, that irritability is accumulated, and therefore *weak* brandy and water is to be preferred as the stimulant, as was employed in this instance

I now ordered more blankets to be added to the former covering.

When respiration ceases in a drowned or suspended person, the power of generating heat is suspended, and the body loses gradually its natural heat, until it be reduced to the temperature of the surrounding medium. During this period, if we attempt to raise the heat to the natural standard by warm pans of coals passed over the bed clothes, or heated bottles or bricks applied to the legs and feet, we are not only disappointed in our expectations, but do an actual injury. Thus if a snake be taken from his autumnal hiding place, and exposed to the sun's rays, or in a warm room, he quickly shew signs of returning life, and even increased powers, but he will die from the experiment; whereas another, gradually stimulated by heat, according to his state, will be restored to full animation. Thus by an ill-judged artificial and continued heat, many destroy the patient they had wished to save. But the lungs being supplied with air, the blood receives the oxygen gas, (oxygen and *caloric in its latent state*) which is diffused to be decomposed, through innumerable arteries and veins, from the centre to the extremities. Thus is the *animal heat* restored, and communicated throughout the system, and with more certainty than from any other mode. The most efficacious method therefore of imparting *heat* is, to excite the generating causes, by renewing respiration, and by placing the patient betwixt blankets, which it escapes as it becomes generated, flannel being a bad conductor of heat. To produce a quicker evolution of *animal heat*, an eighteen gallon cask of oxygen gas was procured, and the air was taken out of the barrel by means of a common bellows, and forced into the lungs of the patient.

Each

Each time of this process, there was an evident improvement for the better. Our patient became less convulsed, and the countenance visibly lost more and more of that ghastly lividness, which was the most alarming symptom. All the attendants noticed, each time, these very salutary changes.

In order to relieve the head, for although the proximate cause of suspended animation is the pressure on the trachea, or wind-pipe, yet, as probably there is *some turgescence* in the brain, owing to former pressure on the jugulars, a dozen leeches were applied to the temples, but not before the powers of life were sufficiently established, and these bled very freely.*

Then also a stimulating enema was thrown up with some tincture of aloes in it, to invite the aorta descendens. Cataplasms were likewise, for the same purpose, applied to the feet.

At night I saw my patient again, greatly restored, but still very insensible; and having got down a draught with valerian, and the volatile tincture of valerian, mixed in some camphor julep and cinnamon water, the cataplasm and blister across the thorax I ordered to be removed; but another was placed along the nape of the neck, when I left him. I was informed by his nurse, that he was restless that night, occasionally convulsed, had some intervals of sleep; but when I saw him in the morning, he was perfectly himself, yet had no recollection of any thing that had passed; the whole narrative seemed to him a dream; he felt sore all over; was very thankful to find himself in existence, and even could smile at what appeared to him a strange event, and which had brought him actually on the very brink of eternity, but without any remembered sensation.

---

### *To the Editors of the Medical and Physical Journal.*

Gentlemen,

WITHOUT eulogium upon the excellency of your publication, and the variety of information it embraces, I beg leave to communicate a Case of Gastritis, which came under

---

* John Hunter says, "I would by all means discourage blood-letting which I think weakens the animal principle, and life itself, consequently lessens both the power and disposition to action."

Philosophical Transactions.

